# Expression and Function of Osteopontin in Vascular Adventitial Fibroblasts and Pathological Vascular Remodeling

**DOI:** 10.1371/journal.pone.0023558

**Published:** 2011-09-19

**Authors:** Xin Jin, Guo-xiang Fu, Xiao-dong Li, Ding-liang Zhu, Ping-jin Gao

**Affiliations:** 1 State Key Laboratory of Medical Genomics and Shanghai Key Laboratory of Vascular Biology, Shanghai JiaoTong University School of Medicine, Ruijin Hospital, Shanghai, China; 2 Laboratory of Vascular Biology, Institute of Health Science, Shanghai Institutes for Biological Sciences (SIBS), Chinese Academy of Sciences (CAS), Shanghai, China; 3 Shanghai Institute of Hypertension, Shanghai, China; 4 Faculty of Biological Science, Institute of Membrane and Systems Biology, University of Leeds, Leeds, United Kingdom; Centro Cardiologico Monzino, Italy

## Abstract

Osteopontin is known to play important roles in various diseases including vascular disorders. However, little is known about its expression and function in vascular adventitial fibroblasts. Adventitial fibroblasts have been shown to play a key role in pathological vascular remodeling associating with various vascular disorders. In this study, we measured activation of Osteopontin and its biological functions in cultured adventitial fibroblasts and injured rat carotid injury arteries induced by balloon angioplasty. Our results showed that angiotensin II and aldosterone increased Osteopontin expression in adventitial fibroblasts in a time- and concentration-dependent manner. MAPKs and AP-1 pathways were involved in Osteopontin upregulation. In addition, Adventitial fibroblast migration stimulated by Angiotensin II and aldosterone required OPN expression. Perivascular delivery of antisense oligonucleotide for Osteopontin suppressed neointimal formation post-injury. We concluded that upregulation of Osteopontin expression in adventitial fibroblasts might be important in the pathogenesis of vascular remodeling after arterial injury.

## Introduction

Osteopontin (OPN) an acidic and secreted glycoprotein plays various roles in bone morphogenesis, cell transformation, immune cell activation, and bacterial resistance [1], [2], [3], [4]. Recently, it has been found that the expression of OPN is involved in vascular inflammatory response [5], [6], [7]. However, the expression and function of OPN in adventitial fibroblasts is unknown.

Recently, there is emerging evidence that adventitial fibroblasts play a vital role in neointimal formation [8], [9], [10], [11], [12]. It is believe that endothelium damage induces the expression of growth factors, cytokines, chemoattractants, which promotes early adventitial activation and neointima formation [13]. Our previous study indicated that TGFβ1 triggered differentiation of vascular adventitial fibroblasts to myofibroblasts and the up-regulation of protein kinase Cα was involved in this differentiation [14]. Recently, we reported that angiotensin II (Ang II), phorbol ester, basic fibroblast growth factor, and vascular endothelial growth factor (VEGF) induced migration of adventitial fibroblasts [12], [15]. Interestingly, we found that Osteopontin augments migratory ability of culture cells from spontaneously hypertensive rats, although the mechanisms are not yet clear[16]. The renin–angiotensin–aldosterone system is now implicated in the development of hypertensive vascular and vascular remodeling disease, there is evidence for aldosterone (ALD) and angiotensin II impair endothelium-related vasodilatation and contribute to inflammation and vascular and cardiac remodeling[17], [18].

Therefore, we hypothesize that OPN is upregulated in vascular advential by renin-angiotensin-aldosterone system, which thus plays an important role in neointima formation. To test this hypothesis, we determined whether the expression of OPN in vascular adventitial fibroblasts was induced by Ang II or ALD and then we investigated the role of OPN in neointima formation using OPN antisense oligo, we also examined the signaling pathways involved in OPN induction in vascular adventitial fibroblasts.

## Results

### 1. OPN expression was regulated by Ang II and ALD in vascular adventitial fibroblasts

**Figure 1:**
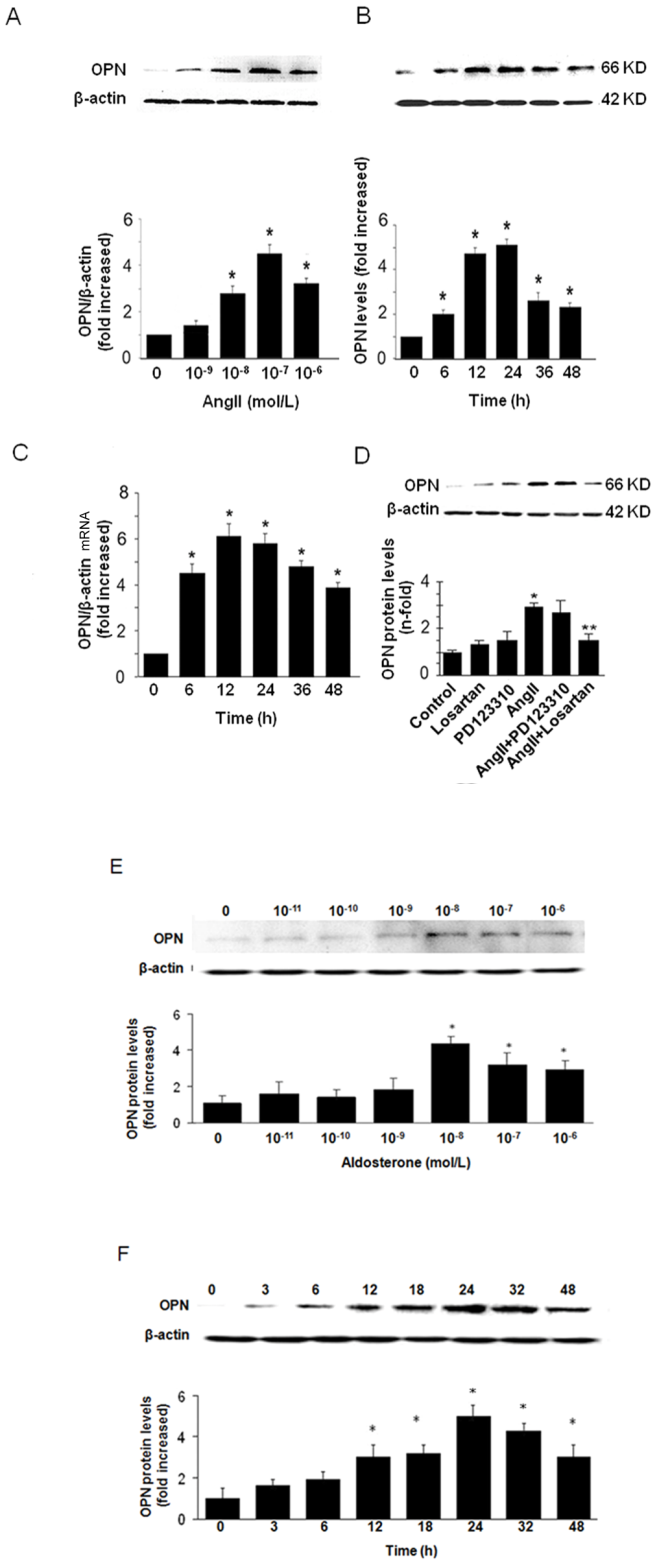
Increase in OPN in adventitial fibroblasts by Ang II and ALD. (A) Ang II-induced expression of OPN protein in a dose-dependent manner. The effect of Ang II on OPN expression was observed at 24 h, the concentration for maximal effect of Ang II was observed at 10^−7^ mol/L. (B) the effects of Ang II on adventitial fibroblasts expression were time-dependent. The effect of Ang II on OPN expression was observed at 10^−7^ mol/L. The maximal effect of Ang II on OPN expression was observed at 24 h. (C) Adventitial fibroblasts were treated with Ang II (10^−7^ mol/L) for varying amounts of time following serum deprivation for 48 h. OPN mRNA levels were quantified by real-time RT-PCR and were normalized to β-actin mRNA levels. (D) The effects of Ang II on OPN expression were blocked by the AT1 receptor blocker losartan, whereas the AT2 receptor blocker PD 123,319 had no effect. The gels represent results of 3 independently performed experiments. (E) ALD -induced expression of OPN protein in a dose-dependent manner. The effect of ALD on OPN expression was observed at 24 h. The concentration for maximal effect of Ang II was observed at 10^−8^ mol/L. (F) the effects of ALD on adventitial fibroblasts expression were time-dependent. The concentration for effect of Ang II was observed at 10^−8^ mol/L. The maximal effect of ALD on OPN expression was observed at 24 h. Normalized data (bar graphs) are presented as mean ± SEM. * P<0.05 vs. adventitial fibroblasts in DMEM alone (control), ** P<0.05 vs. adventitial fibroblasts in Ang II alone.

To investigate the effects of Ang II and ALD on OPN expression, adventitial fibroblasts were treated with various doses of Ang II and ALD. First, we examined the effect of Ang II on the expression of OPN. As shown in Fig. 1A, Ang II induced OPN expression in a dose-dependent manner, with the maximal effect observed at 10^−7^ mol/L Ang II. Ang II also induced the OPN expression in a time-dependent manner, with the maximal effect at 24 h (Fig. 1B). We next examined whether the increase in OPN protein expression by Ang II resulted from the induction of OPN mRNA expression, We found that Ang II time-dependently induced OPN mRNA in adventitial fibroblasts as assessed by real-time reverse transcription polymerase chain reaction (RT-PCR) (Fig. 1C), OPN mRNA was significantly increased within 6 h, peaked by 12 h, and remained up to 48 h. To further determine the role of Ang II receptors in OPN expression, adventitial fibroblasts were pretreated with the specific angiotensin II type 1 (AT1) receptor blocker losartan (10^−4^ mol/L) or the angiotensin II type 2 (AT2) receptor blocker PD 123319 (10^−4^ mol/L) for 30 min, and then the cells were exposed to Ang II (10^−7^ mol/L) for 24 h. We found that the AT1 receptor blocker losartan but not AT2 receptor blocker PD 123319 significantly blocked the effect of Ang II on OPN protein expression (Fig. 1D). These indicate that Ang II induces OPN expression through AT1 receptor.

Similarly, ALD also induced OPN expression in a dose-dependent manner, with the maximal effect observed at 10^−8^ mol/L concentration of ALD (Fig. 1E). ALD also induced the OPN expression in a time-dependent manner, with the maximal effect of ALD observed at 24 h (Fig. 1F).

### 2. Mitogen-activated protein kinases (MAPKs) were involved in OPN Expression

**Figure 2:**
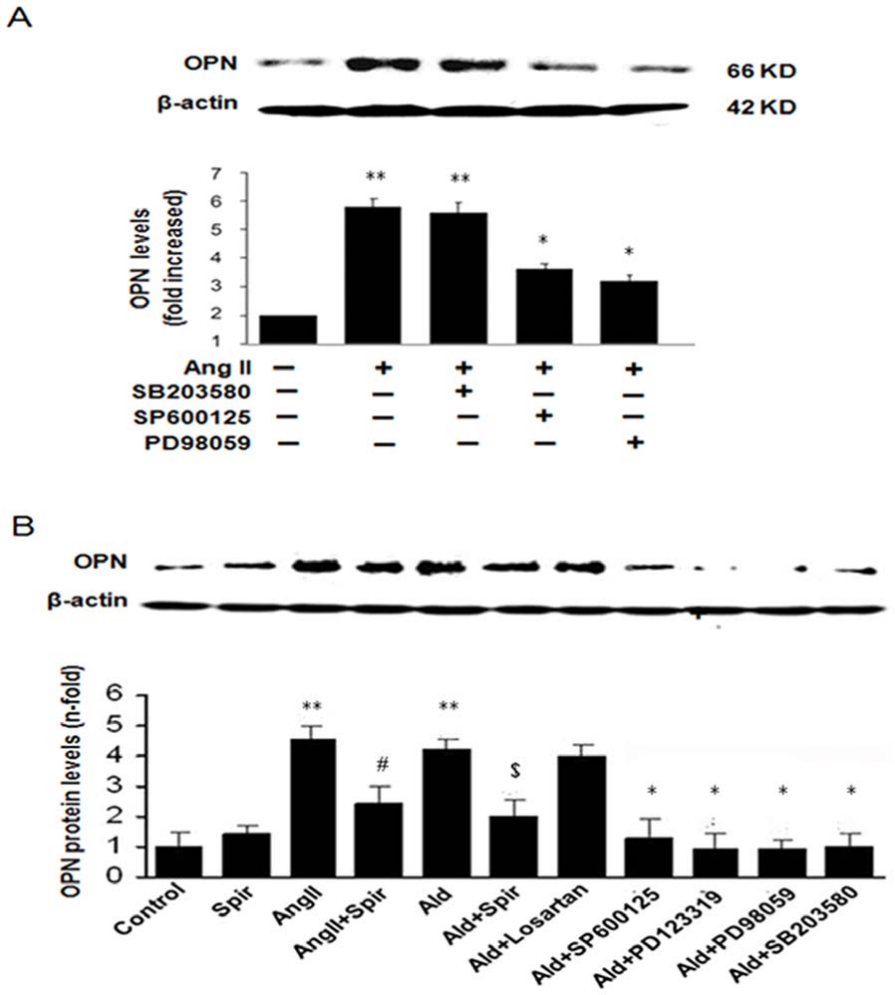
MAPK pathways were involved in OPN expression in Adventitial fibroblasts. (A) Cells were preincubated with 20 µM PD98059, SB203580, SP600125 for 1.5 h and then treated with 10^−7^ mol/L Ang II for 12 h. (B) Cells were treated with ALD(10^−8^ mol/L), Ang II (10^−7^ mol/L) alone or along with Spir, losartan or PD 123,319 for 24 h. Then cells were preincubated with 20 µM PD98059, SB203580, SP600125 for 1.5 h and then treated with 10^−8^ mol/L ALD for 24 h. OPN expression was determined by Western blotting analysis. The gels represent results of 3 independently performed experiments. Normalized data (bar graphs) are presented as mean ± SEM. * P<0.05 vs. adventitial fibroblasts in Ang II and ALD alone. ** *P*<0.05 vs. adventitial fibroblasts in DMEM. # *P*<0.05 vs. adventitial fibroblasts in Ang II. $ *P*<0.05 vs. adventitial fibroblasts in ALD.

We previously showed that Ang II activates MAPKs in adventitial fibroblasts [19]. Therefore, we examined whether the MAPK activation was involved in Ang II–induced OPN expression in adventitial fibroblasts. Pretreatment with a JNK1/2 inhibitor SP600125 (20 µM), p42/p44 MAPK inhibitor PD98059 (20 µM) for 1.5 h significantly blocked Ang II -induced OPN protein expression (Fig. 2A). In contrast, pretreatment with a p38 MAPK inhibitor SB203580 did not blocked Ang II-induced OPN expression. These results suggest that Ang II induced OPN expression via a JNK and p42/p44 signaling pathway in adventitial fibroblasts.

Differently, pretreatment with a JNK1/2 inhibitor SP600125 (20 µM), p42/p44 MAPK inhibitor PD98059 (20 µM), p38 MAPK inhibitor SB203580 for 1.5 h significantly blocked ALD-induced OPN protein expression (Fig. 2B). We also found ALD antagonist spironolactone (Spir) not only inhibit ALD-induced OPN protein expression but also blocked Ang II -induced OPN protein expression by AT2 receptor. Since Ang II induce ALD secretion by AT2 receptor in some cell type [20], [21], Ang II and ALD synergistically induced OPN protein expression in adventitial fibroblasts, and Spir block the effect of ALD.

### 3. AP-1 was involved in OPN Gene Upregulation

**Figure 3:**
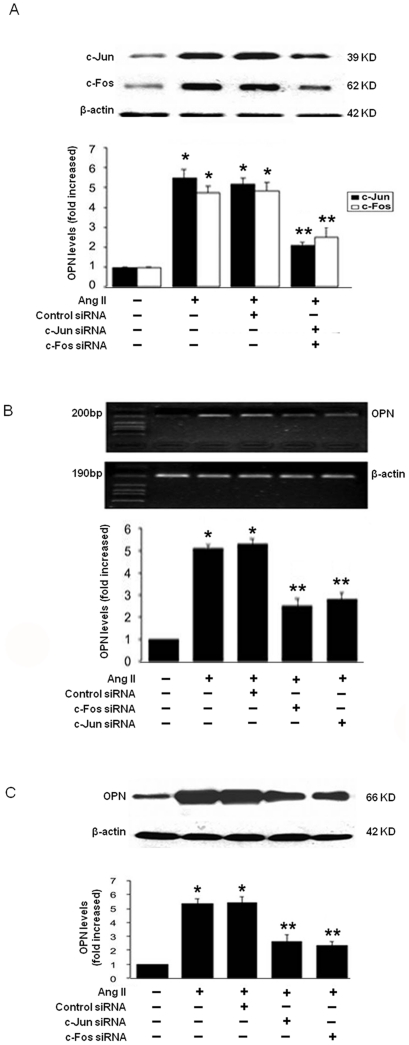
Upregulation of OPN protein(C) and mRNA (B) expression mediated through AP-1 (c-Fos/c-Jun) pathway in Adventitial fibroblasts. (A) Adventitial fibroblasts were transfected with c-Fos, c-Jun siRNA or Control-siRNA, and then stimulated with Ang II (10^−7^ mol/L) for 1 h. The protein of c-Jun or c-fos was assayed by Western blot. (B) Adventitial fibroblasts were transfected with c-Fos, c-Jun siRNA or Control-siRNA, and then stimulated with Ang II (10^−7^ mol/L) for 10 h. The mRNA of OPN was assayed by RT-PCR. (C) Adventitial fibroblasts were transfected with c-Fos, c-Jun siRNA or Control-siRNA, and then stimulated with Ang II (10^−7^ mol/L) for 12 h. The protein of OPN was assayed by Western blot. (D)

To define the transcription factor that mediates Ang II-induced OPN expression, we focused on AP-1 (c-Fos/c-Jun), we found that cells transfected with c-Fos or c-Jun siRNA significantly reduced c-Fos or c-Jun proteins, respectively (Fig. 3A). Downregualtion of c-Fos or c-Jun by siRNA attenuated Ang II-induced OPN expression at the levels of both mRNA (Fig. 3B) and protein (Fig. 3C). This demonstrates that AP-1 (i.e. c-Fos and c-Jun) is essential for mediating Ang II-induced OPN expression in adventitial fibroblasts.

**Figure 4:**
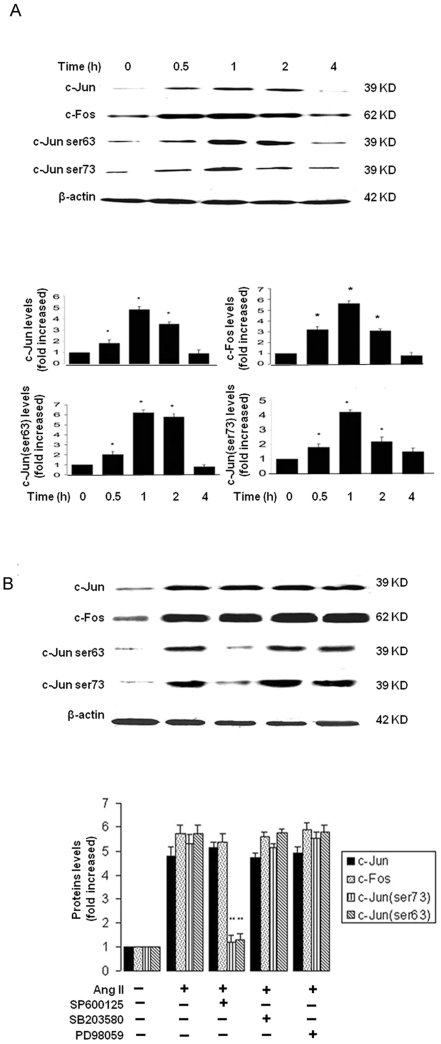
Ang II induced AP-1 activity. (A) Adventitial fibroblasts were treated with Ang II (10^−7^ mol/L) at the indicated time, the protein expression of c-Jun and c-Fos and the phospharylation levels of c-Jun (ser63, ser73) were identified. (B). Effect of PD98059, SB203580, SP600125 on Ang II-induced AP-1 expression and phospharylation, cells were pretreated with 20 µM PD98059, SB203580, SP600125 for 1.5 h and then incubated with 10^−7^ mol/L Ang II. The intensities of bands were quantified. The gels represent results of 3 independently performed experiments. Normalized data (bar graphs) are presented as mean ± SEM. * P<0.05 vs. adventitial fibroblasts in DMEM alone. ** P<0.05 vs. adventitial fibroblasts in Ang II alone.

We next determined if Ang II induces c-Fos/c-Jun expression in adventitial fibroblasts. As shown in Fig. 4A, Ang II markedly induced c-Jun/c-Fos protein, with peaked within 1 h and remained up to 2 h as assessed by Western blot. To further determine whether Ang II-stimulated JNK activation leads to c-Jun phosphorylation, we found that Ang II stimulated the c-Jun phosphorylation, with peaked at 1 h (Fig. 4A). This was inhibited by JNK inhibitor (SP600125) but not by ERK1/2 inhibitor (PD98059) (Fig. 4B).

These results indicate that Ang II induced an immediate early gene c-Jun expression and also activation through a JNK-dependent pathway, thereby leading to OPN expression in adventitial fibroblasts.

**Figure 5:**
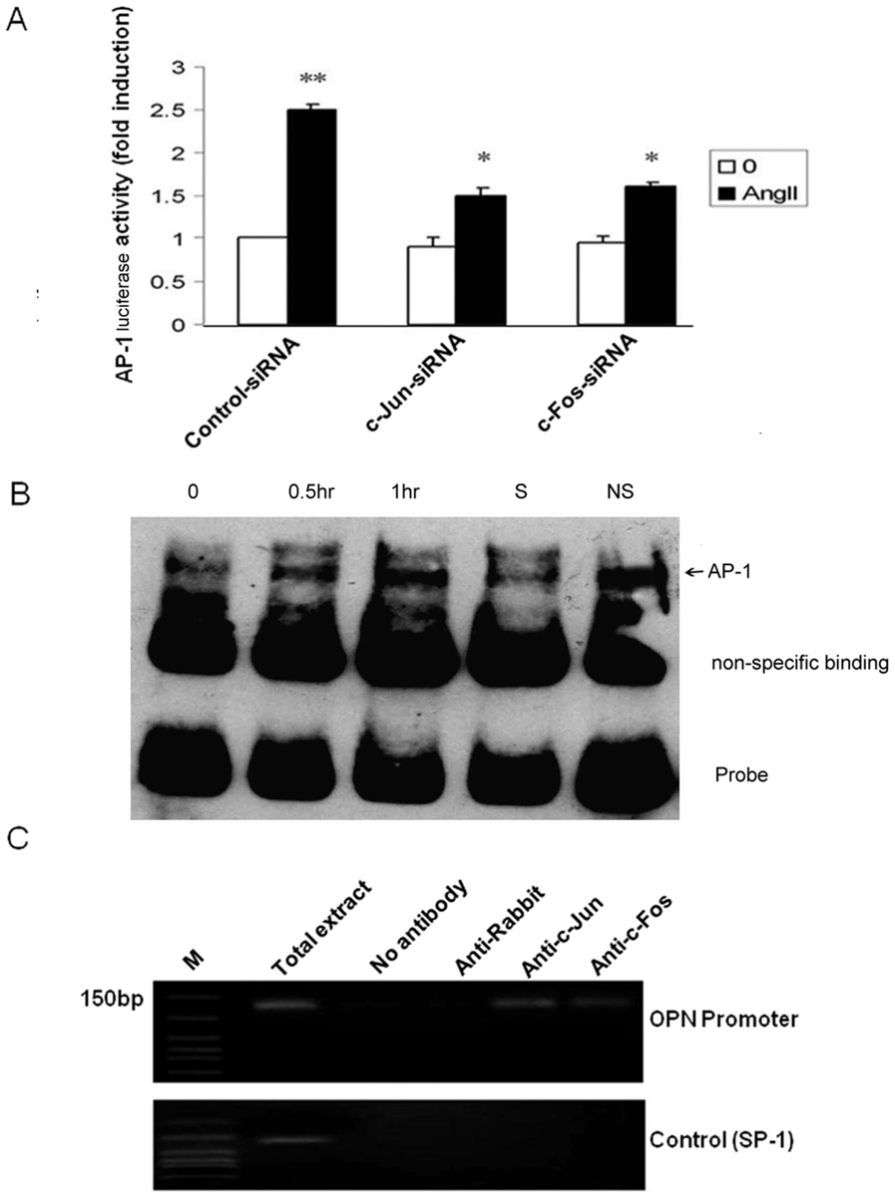
Ang II induced c-Jun and c-Fos DNA binding activity. (A) Adventitial fibroblasts were co-transfected with pAP-1-Luc and 0.2 µg of β-Gal luciferase reporter control vector in the absence or presence of siRNA for c-Jun or c-Fos. Twenty-four hours after transfection, the cells were treated with Ang II (10^−7^ mol/L) for 1 h, the cells were lysed and luciferase activities were measured. (B) Adventitial fibroblasts were treated with 10^−7^ mol/L Ang II at different time and the nuclear extracts were prepared. Gel-shift assays were performed. The specific shifted AP1 components are indicated by arrows. (C) Cells were cross-linked with 1% formaldehyde; nuclear lysate was then prepared. After precipitation with different anti-c-Jun, c-Fos antibodies or normal goat serum, the OPN promoter regions were amplified by PCR, respectively. The gels represent results of 3 independently performed experiments. * P<0.05 adventitial fibroblasts were transfected with siRNA for c-Jun or c-fos vs. control-siRNA, ** P<0.05 adventitial fibroblasts in Ang II vs. DMEM alone.

To definitively determine the role of AP-1 in regulation of OPN transcription, we first determined whether AP-1-dependent transcriptional activity was induced by Ang II in adventitial fibroblasts using pAP-1-Luc reporter construct and luciferase analysis. As shown in Fig. 5A, Ang II enhanced AP-1-dependent transcriptional activity, which was inhibited by c-Fos or c-Jun siRNA.

We next determined whether Ang II induced the binding of c-Jun and c-Fos to the OPN Promoter in vitro by gel shifting analysis. We designed the biotin labeled AP-1 probes (5′- TTC CGGCTGACTCATCAAGCG - 3′ biotin) based on the AP-1 binding site sequence (-76) located in the mouse OPN promoter. Nuclear extracts from adventitial fibroblasts treated with Ang II for indicated time were incubated with AP-1 probe, and AP-1 DNA complex was detected by gel electrophoresis. We found that Ang II induced AP-1 DNA complex formation in a time dependent manner (Fig. 5B). The specificity of the AP-1 binding was determined by competition experiments with excess of unlabeled oligonucleotide containing the AP-1 binding site (Fig. 5B, lane4) Furthermore, we determined whether Ang II induced AP-1 binding to OPN promoter in vivo using chromatin immunoprecipitation (ChIP)-PCR. Chromatin was immunoprecipitated using an anti-c-Fos or anti-c-Jun antibody, and the OPN promoter region was amplified by PCR using a pair of primers encompassing an AP-1 binding site (-76) in OPN promoter. As shown in Fig. 5C, in vivo binding of c-Jun/c-Fos to the OPN promoter was increased upon Ang II stimulation. Together, these results demonstrate that Ang II indeed induced the binding activity of c-Fos and c-Jun to the AP-1 site in the promoter region of OPN gene.

### 4. OPN is critical for Ang II and ALD-induced adventitial fibroblast migration

**Figure 6:**
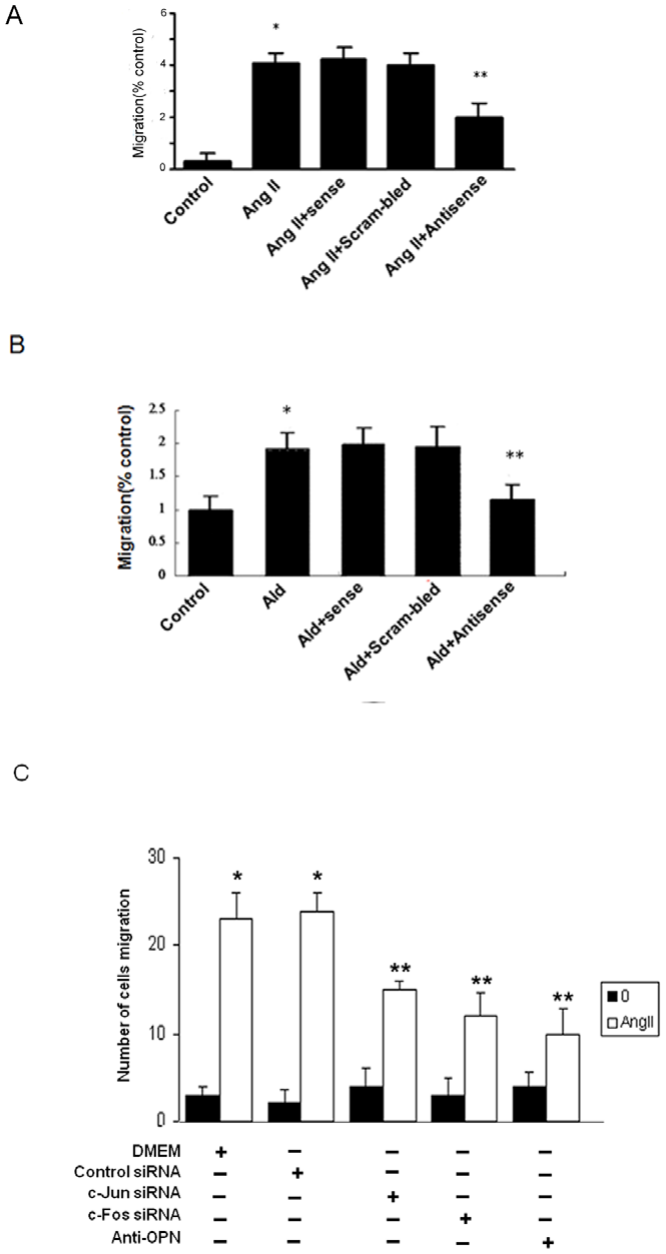
Effect of anti-sense or neutralized antibody for OPN and AP-1-siRNA on the migration of Adventitial fibroblasts. (A) Adventitial fibroblasts were treated with Ang II (10^−7^ mol/L) for 8 h and subsequently studied cells migration by using transwell apparatus. The increased migration was blocked by treatment with anti-sense for OPN. (B) Adventitial fibroblasts were treated with ALD (10^−8^ mol/L) for 8 h and subsequently studied cells migration by using transwell apparatus. The increased migration was blocked by treatment with ant-sense for OPN. (C) Tranfected with siRNA for c-Jun or c-fos or pretreated with neutralized antibody for OPN then adventitial fibroblasts were treated with Ang II (10^−7^ mol/L) for 8 h and subsequently studied cells migration by using transwell apparatus. Each bar represents mean ± SE of cell migrated per high- powermicroscope for three independent experiments. * P<0.05 vs. adventitial fibroblasts in DMEM alone. ** P<0.05 vs. adventitial fibroblasts in Ang II alone.

Our previous study has shown that Ang II and ALD increased adventitial fibroblasts migration [12], to determine if the migration of adventitial fibroblasts is OPN-dependent, migration experiments with Ang II and ALD were performed. As shown in Fig 6A and B, Ang II or ALD significantly increased migration measured by transwell chamber. OPN anti-sense significantly attenuated Ang II or ALD-induced adventitial fibroblasts migration, while OPN sense or scramble sense cannot inhibit ALD or Ang II-induced adventitial fibroblasts migration, demonstrating that OPN is essential for Ang II or ALD-induced migration in adventitial fibroblasts. To determine if the migration of adventitial fibroblasts is also AP-1-dependent, migration experiments with Ang II were performed in the presence of siRNA of c-Fos or c-Jun. As shown in Fig. 6C, Ang II significantly increased migration measured by transwell chamber. C-Fos or c-Jun siRNA significantly attenuated Ang II-induced adventitial fibroblasts migration, demonstrating that AP-1 (i.e. c-Fos and c-Jun) is essential for Ang II-induced migration in adventitial fibroblasts. We also found OPN neutralize antibody attenuated Ang II-induced adventitial fibroblasts migration.

### 5. OPN was involved in neointimal hyperplasia after balloon injury

**Figure 7:**
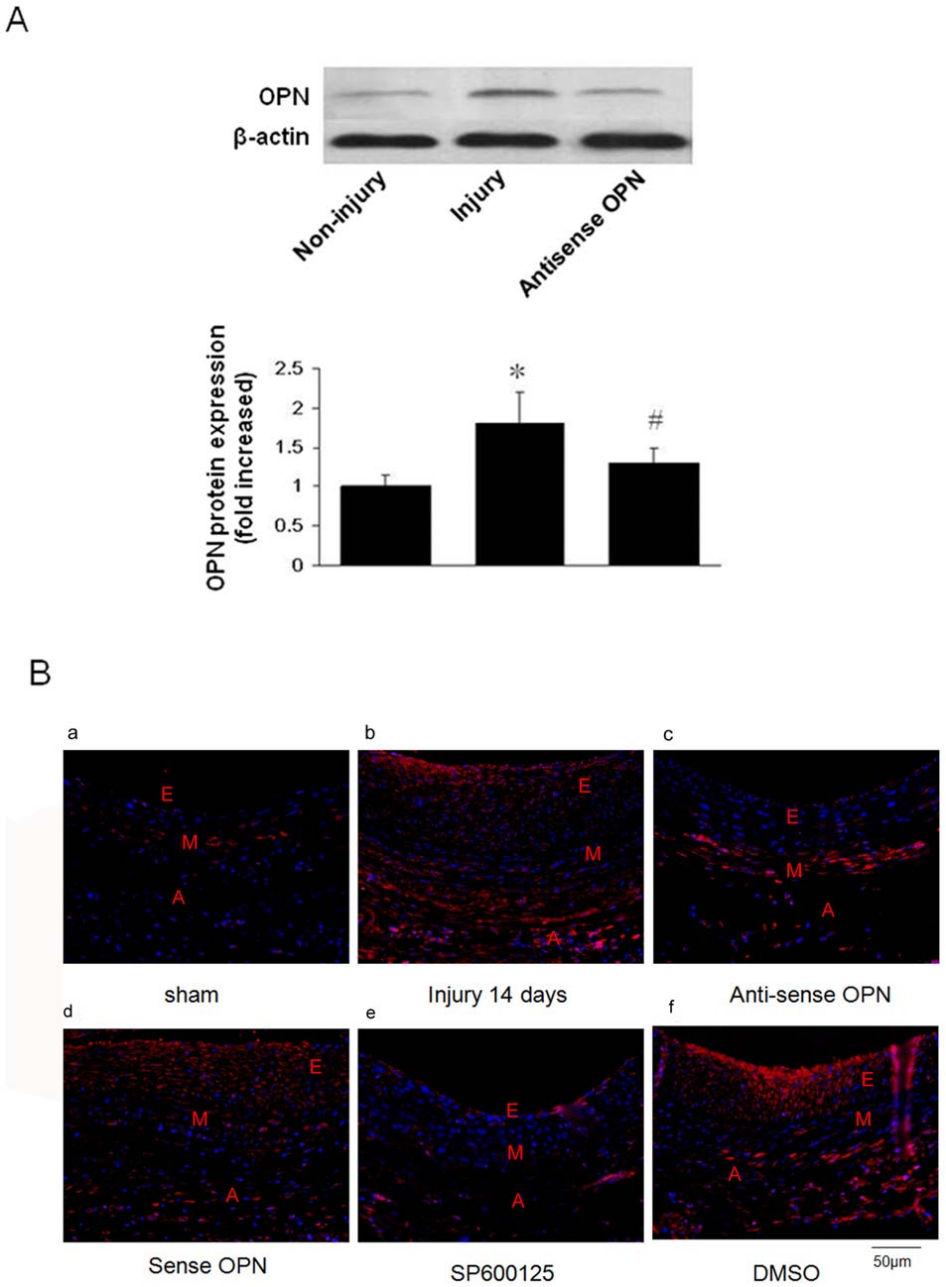
Expression of OPN after rat carotid operation. (A) Expression of OPN after rat carotid injury. Adventitial separated from non-Injured adventitial arteries, injured adventitial arteries or injured adventitial arteries for antisense OPN were assayed by westernblotting 28 days after carotid injury. (B) OPN expression is demonstrated immunofluorescently in arterioc wall. **a**, Sham-operation ; b,operation injury 14 days; c, operation+anti-sense OPN treated; d, operation+sense OPN treated; e, operation+sp600125; f, operation+DMSO. Scale bars represents 50 µm (E, Endothelium; M, Medial; A, Adventitial).

We determine the role of OPN in neointimal hyperplasia after balloon injury using perivascularly applied OPN antisense oligo. We first examined OPN expression by Western blotting analysis. Taking adventitial layer from vascular layers, as shown in Fig. 7A, balloon injury dramatically increased the OPN protein level in the adventitial layer of rat carotid arteries, which is significantly attenuated in the arteries treated with OPN antisense oligo. This indicates that perivascular application of OPN antisense oligo effectively prevented OPN upregulation after injury.

We then examined the expression of OPN in both neointima and adventitia of rat carotid arteries after balloon injury by immunofluorescent assay. As shown in Fig 7B (panel b versus panel a), in compare to sham-operation group, OPN immunostaining increased in the neointima, media, and adventitia on day 14, which was attenuated in the adventitial layers of the arteries treated with OPN antisense oligo compared with sense oligo (Fig 7B, panel c versus panels d and b). To monitor the effect of MAPKs on OPN expression in vivo, carotid arteries were treated with SP600125 by adventitial administration in vivo, as shown in Fig 7B(panel e versus panels b and f), SP600125 delivery to the adventitia inhibited markedly OPN expression in vivo after balloon catheter injury compared to dimethyl sulfoxide (DMSO) group.

**Figure 8:**
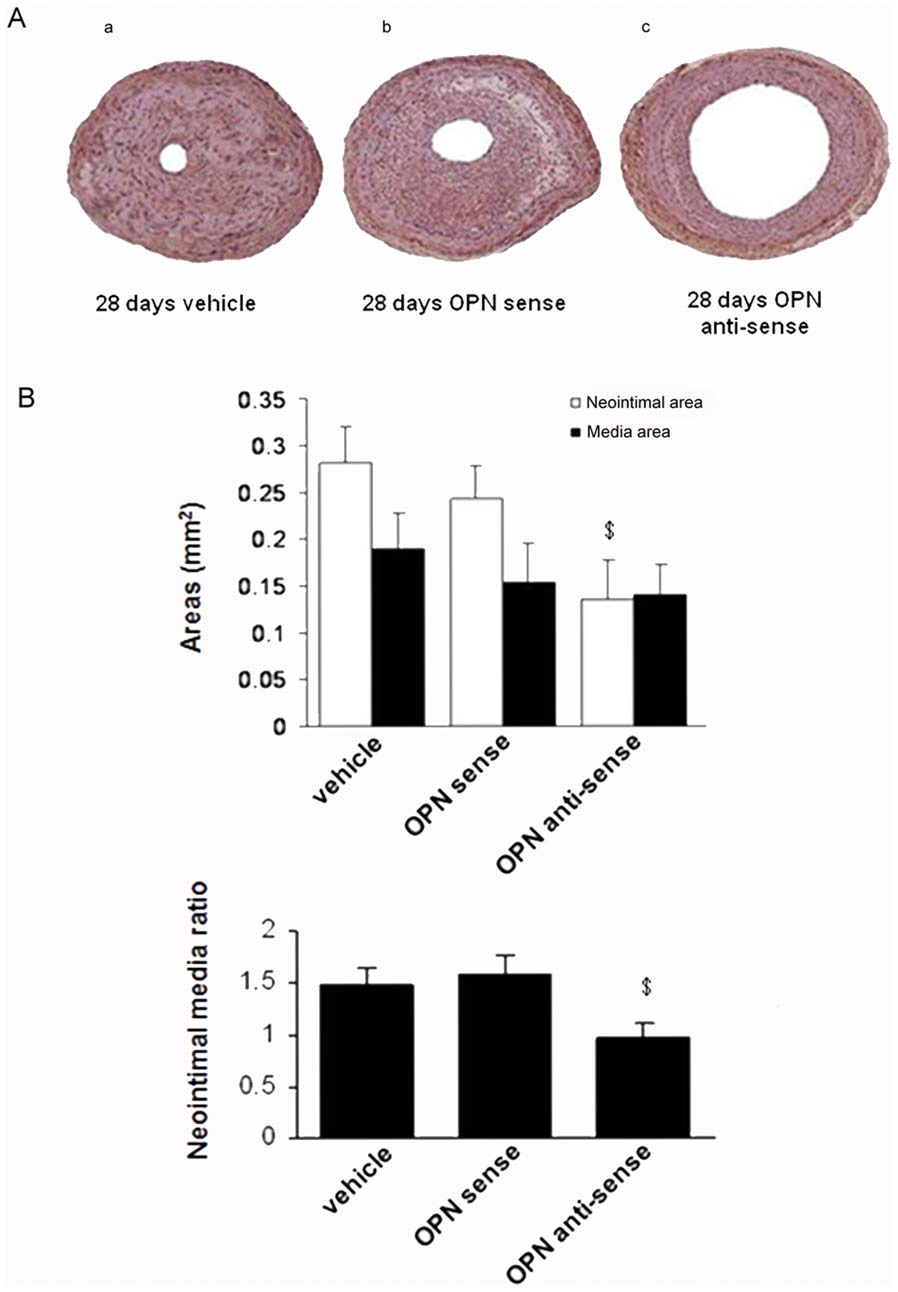
OPN antisense inhibited neointima formation in injured carotid arteries. (A) Representative light micrographs of common carotid arteries from Veh-, sense-OPN, and antisense-OPN treated rats at day 28 after balloon injury. Arterial sections were stained with HE stain. (B) Effects of administration of antisense-OPN on neointima area and neointima/media ratio of injured carotid arteries in rats at 28 days after carotid artery balloon injury. The results are taken from 3 rats per group and represent 3 independently performed experiments. Data are shown as mean+SEM. $ *P*<0.05 vs. Veh group and sense-OPN. * *P*<0.05 vs. normal arterial adventitial layers. # *P*<0.05 vs. arterial adventitial layers at day 28 after balloon injury.

We then evaluated the effects of OPN antisense oligo on vascular remodeling. As shown in Fig. 8A, neointima formation at 28 days after injury was significantly reduced in vessels treated with OPN antisense oligo compared with sense oligo (Fig. 8A, panels c versus panels a and b). The quantitative data were shown in Fig. 8B. The neointimal area at 28 days after the injury was 0.12±0.06 mm^2^ in antisense-OPN-treated arteries, and significantly smaller than the corresponding area in injured arteries treated by sense-OPN or vehicle (0.27±0.07 mm^2^, *p*<0.05). The antisense-OPN also decreased the I/M area in arteries compared with sense-OPN or vehicle treated arteries (0.67±0.31 vs. 1.57±0.34, *p*<0.05).

### 6. OPN influence fibroblast function through MAPKs pathway

**Figure 9:**
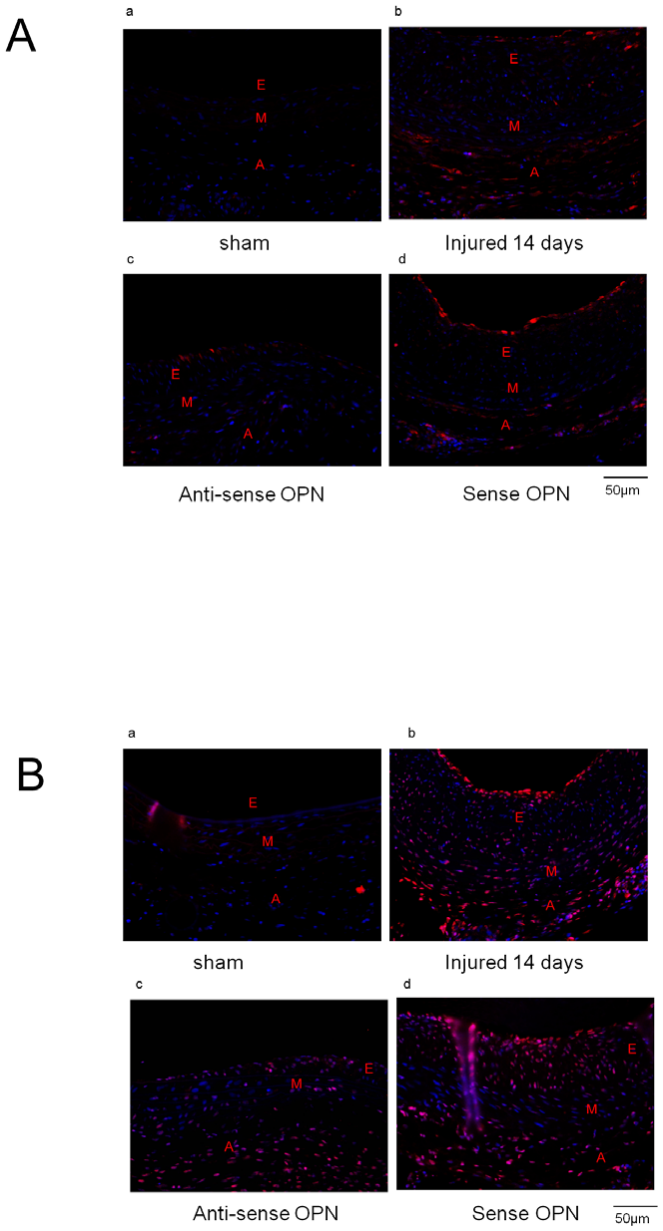
Effects of OPN on MAPKs. Representative sections, harvested 14 days following balloon angioplasty, immunostained for phospho-ERK1/2 (A), phospho-p38 (B). **a**, Sham-operation ; b, operation injury 14 days; c, operation+anti-sense OPN treated; d, operation+sense OPN treated; Scale bars represents 50 µm (E, Endothelium; M, Medial; A, Adventitial).

We then explored the potential molecular pathways involved in fibroblast activation by OPN. As shown in Fig 9A and B (panels b versus panels a), compared with the sham-operation group, the aortic wall at the lesion sites showed enhanced expression of phospho-ERK1/2 and phospho-p38. In contrast, the expression level of phospho-ERK1/2 was reduced at both the adventitial and neointimal layers of the arteries treated with OPN antisense oligo compared with sense oligo and catheter injury group (Fig 9A, panels c versus panels b and d). Similarly, the expression level of phospho-p38 was lower in the neointimal layer of the arteries treated with OPN antisense oligo compared with sense oligo and catheter injury group (Fig 9B, panel c versus panels b and d).

## Discussion

Several previous studies have shown that OPN plays an important role in cardiovascular disease, such as myocardial ischemia/hypoxia, cardiac valve formation and vascular inflammation, and remodeling [22], [23], [24], [25], [26], [27], [28], [29], [30], [31], [32]. While OPN regulation has been studied in several different cell types [32], [33], [34], [35], its role and regulation in vascular adventitial fibroblasts have never been evaluated.

In the present studies we used the in vivo balloon-injured rat carotid artery model and demonstrated OPN expression in adventitial after injury. Adventitial transduction of antisense oligo targeting OPN attenuated injury-induced OPN upregulation, inhibited adventitial fibroblasts migration, and prevented neoinitma formation. In addition, MAPK and AP-1 pathways were involved in the regulation of OPN. Based on these results, we conclude that growth factors such as Ang II and ALD mediated regulation of OPN are an important mechanism responsible for regulation of adventitial fibroblast migration and subsequent neointima formation.

Over the past decade increasing evidence suggests an important role for the adventitia in certain physiological and pathological conditions [36], [37], [38], [39]. Our previous studies have suggested that perivascular transfer of exogenous genes primarily affected adventitial but not vascular smooth muscle cells or endothelial cells [40]. OPN antisense oligo applied perivascularly reduced neointima formation, with also supports an important role of adventitial in regulating neointimal hyperplasia and vascular remodeling. In addition, compared with other intraluminal catheter delivery approach, perivascular transfer has the advantages of preventing rapid development of neointimal lesions.

Upregulation of OPN has been described in models of cardiac hypertrophy and heart failure, and MAPK and AP-1 appear to be important. In our study, we firstly found that JNK activation is required in OPN expression by Ang II and ALD in aortic adventitial fibroblasts.

The AP-1 transcription factor consists of a large set of dimers containing Jun, FOS, and ATF families of proteins. They contain transactivation domains, DNA-binding domains, and leucine zippers [41], [42], [43]. Sharma et al have shown Ang II induce c-jun and c-fos mRNA expression [44]. In this study, we demonstrate that Ang II increased c-Jun phosphorylation and thus activation, which was attenuated by pretreatment with inhibitors of JNK. Interestingly, inhibitors of JNK do not inhibit Ang II-induced c-Fos expression while it inhibits Ang II-induced migration; this result is consisted with other studies [45], [46]. Our data also showed that Ang II stimulates AP-1 transcription activity (either c-Jun or c-Fos), which acts as an important mediator in OPN upregulation and adventitial fibroblasts migration.

To further examine whether AP-1 binds to AP-1 sites in the promoter region of OPN gene, the binding activity of AP-1 was determined by EMSA and ChIP-PCR assay. Our data showed that Ang II up-regulates OPN expression through stimulating c-Jun and c-Fos binding to the -76AP-1 site.

OPN-dependent fibroblast activation in our balloon injury model may be via both a direct and an indirect effect of OPN on adventitial fibroblasts. Evidence in support of a direct role for OPN is that OPN anti-sense attenuated Ang II or ALD-induced adventitial fibroblasts migration, as well as our previous studies that demonstrate a direct effect for OPN on fibroblasts in vitro including enhanced migration and proliferation[16]. Other studies have also confirmed the direct activatory role for OPN on cardiac fibroblasts [47]. OPN may also activate vascular fibroblasts indirectly via activating MAPK pathway. Our findings support this: first, the injury aortic wall increase the expression of phospho-ERK1/2, and phospho-p38, while OPN antisense treated injury rats show reduced expression of phospho-ERK1/2 and phospho-p38 in aortic wall. Second, activation of the MAPK pathway has an impact on the vascular remodeling in vivo, for MAPK pathway have many targets in the downstream, including connective tissue growth factor, VEGF, and many others. These moleculars may have an impact on the vascular remodeling. We use MAPKs pathway inhibitor also inhibit the neointimal hyperplasia in vivo (Figure S1). Until now, which downstream target remain unknown, it requires extensive investigation.

Recent evidence indicates the presence of progenitor cells in the adventitial [48], [49], [50], [51], [52], [53], [54], [55], [56]. Kaplan et al [57] show that Flt-1 was used to identify progenitors; our previous study showed that Flt-1 expression was significantly increased in the neointima and adventitia in injured rat carotid arteries [15]. Many other studies showed osteopontin have an impact on the progenitor activities such as proliferation, migration, and angiogenesis [58], [59], [60]. Together, these data indicate another potential mechanism for the role of OPN in vascular remolding: stem cells could be induced by OPN at adventitia after injury and migrate to the neointima, which contribute to the neointimal hyperplasia response to injury.

In summary, our in vitro and in vivo studies demonstrated an increased expression of OPN in adventitial fibroblasts in response to growth factors such as Ang II and ALD. By using antisense oligo of OPN, we indicated that OPN promotes adventitial fibroblasts migration in vitro and contributes to vascular remodeling after balloon injury in vivo. Our data also suggest that OPN may serve as potential candidates for treating neointimal hyperplasia via AP-1 signaling.

## Materials and Methods

### 1. Materials

The study was approved by the Institutional Review Board of Shanghai Institutes for Biological Sciences of Chinese Academy of Sciences. All experimental protocols were approved by the Animal Care and Use Committee of Shanghai Institutes for Biological Sciences of Chinese Academy of Sciences, the approval numbers for this study is 2003136. Ang II and PD 123319 were obtained from Sigma-Aldrich (St. Louis, Missouri, USA). Losartan was kindly provided by Merck (Rahway, New Jersey, USA). Anti-c-Jun, anti-c-Fos, anti-Phospho-c-Jun (Ser63), anti-Phospho-c-Jun (Ser73) antibodies were purchased from Cell Signaling Technology (Cell Signaling, CA, USA), β-Actin was purchased from Sigma– Aldrich (St. Louis, Missouri, USA). The neutralizing OPN antibody was purchased from R&D Systems (Minneapolis, MN, USA). SiRNA-c-Jun, siRNA-c-Fos was purchased from Santa Cruz (Santa Cruz, CA, USA).

### 2. Cell culture

The isolation and culture of adventitial fibroblasts from thoracic aorta of 12–14-week-old male Sprague–Dawley rats were similar to previously described [61], [62]. Briefly, thoracic aortas were removed and stripped of perivascular connective tissue; the media and endothelium were separated from the adventitia. Then the dissected adventitia was cut in to pieces and planted on dishes. These cells were characterized by positive staining for vimentin, and negative staining for α-SM-actin as described previously [14].

### 3. Synthesis of Antisense Oligonucleotides

Phosphorothioate-derived oligodeoxynucleotides was chemically synthesized by MWG Biotech AG (Germany) and was comprised of the following sequences: OPN antisense 5′-AACCACTGCCAGTCTCAT-3′, OPN sense: 5′-ATGAGACTGGCAGTGGTT-3′, OPN scramble sense: 5′-AACTACTATCAGTCTCGT-3′. The OPN sense oligodeoxynucleotide sequence comprised the first five codons of rat OPN mRNA, and antisense OPN oligodeoxynucleotides comprised the complementary sequence.

### 4. Western blotting

Whole cell extracts were prepared essentially as before [42]. The samples were then resolved on polyacrylamide gels containing SDS and transferred to a nitrocellulose membrane by electroblotting. The membrane was incubated in blocking buffer (TBS Containing 5% skim milk and 0.1%Tween20) for 2 h, followed by incubation with the primary antibody diluted in the same buffer. The specific secondary antibody was detected using peroxidase-conjugated anti-IgG at 1∶2000. Relative proteins were detected by the supersignal chemiluminescence system (ECL, Pierce) followed by exposure to autoradiographic film.

### 5. Electrophoresis mobility shift assay (EMSA)

For EMSA (LightShiftTM Chemiluminescent EMSA Kit, Pierce), 5 µg of nuclear extract was incubated with 2 nmol/L of the end-labeled with biotin double-stranded oligonucleotide probes in reaction buffer for 20 min at room temperature. Samples were resolved on a nondenaturing 4% polyacrylamide–2% glycerol gel, transferred to BiodyneR B Nylon membrane, avidin-HRP to probes, and visualized and quantitated with a PhosphorImager. All the double stranded probes were synthesized as follows: for the binding sites of AP-1 in the OPN promoter: 5′-CAA AAC CAG AGT GGG GAG TG-3′(sense) and 5′-CAA AGC CAA GGA TGC TGA-3′(anti-sense).

### 6. Migration assays

Evaluation of cell migration utilized a transwell cell culture chamber inserts with 8.0-um pores (Corning Costar, Cambridge, MA, USA) in 24-well plates as described previously [15].

### 7. Transient Transfection and Luciferase Assay

Transfections were performed with the SuperFect transfection reagent (Qiagen, Hilden, Germany) method. Luciferase activity was detected with a luciferase assay kit (Promega Corporation, Madison, WI). Transfection of siRNA duplex was done following the manufacturer′s protocol described at Santa Cruz Biotechnology, Inc. (Santa Cruz, CA, USA).

### 8. Chromatin Immunoprecipitation Assay

ChIP assays were performed using the ChIP Assay Kit (Millipore, Temecula, CA) and protocol described at www.millipore.com (17-611). Briefly, quiescent adventitial fibroblasts treated by 10^−7^ mol/L Ang II for 1 hour, cells were cross-linked with 1% final concentration of formaldehyde at 37°C for 10 min before harvest. The DNA was then isolated and sheared by sonication. The proteins of interest were immunoprecipitated along with the DNA to which it was bound using antibodies specific for IgG, c-Jun and c-Fos (upstate), and then the cross-links were reversed. Then, PCR was performed using the OPN gene specific primers for AP-1 binding region, sense 5′ - CAAAACCAGAGTGGGGAGTG - 3′ and antisense 5′ – CAAAGCCAAGGATGCTGA - 3′ primers. The Sp-1 gene fragment control was amplified using 5′- AGAACCGCACAGTCTCTGGT-3′ and 5′- GGGACAGCTTGCTGGAGTAG -3′ primers.

### 9. Quantitative Real-Time RT-PCR

OPN mRNA expression levels were determined by quantitative real-time RT-PCR. Briefly, total RNA was isolated from adventitial fibroblasts stimulated with and without Ang II, using RNAzol reagent (invitrogen).

The real-time PCR was performed using SYBR Green Master Mix and the ABI PRISM 7700 Sequence Detection System (Applied Biosystems, Foster City, CA USA) according to the manufacturer's recommendations. The primer sequences used to amplify β-actin mRNA were 5′ -CCA GGC ACC AGG GCG TGA TG-3′ (sense) and 5′-CGG CCA GCC AGG TCC AGA CG- 3′ (anti-sense); OPN mRNA 5′-CAGTCGATGTCCCTGACGG-3′ (sense) and 5′-GTTGCTGTCCTGATCAGAGG-3′ (anti-sense). Relative levels of OPN mRNA expression were normalized in all the samples analyzed with respect to the levels of β-actin amplification.

### 10. Perivascular delivery of antisense-OPN and SP600125 following rat carotid artery balloon injury

Male 12–14-week-old Sprague–Dawley rats weighing approximately 350–400 g were used in the present study (6–8 rats per group). Antisense-OPN or sense-OPN (as a negative control) was suspended in pluronic F127 gel (Sigma; 20% w/v in sterile saline). Immediately after the injury procedure, 200 µl of pluronic gel containing the adenovirus was applied to the adventitial surface of the artery. After solidification of the gel, the wound was closed and the animals were allowed to recover from the anesthesia. The rats were killed within 28 days after the balloon injury and their common carotid arteries were excised for histological or Western blot analyses.). For SP600125, 100 mmol/L SP600125 and 1% DMSO (as a negative control) was suspended in pluronic F127 gel (Sigma; 20% w/v in sterile saline). Immediately after the injury procedure, 200 µl of pluronic gel containing the adenovirus was applied to the adventitial surface of the artery. After solidification of the gel, the wound was closed and the animals were allowed to recover from the anesthesia. The rats were killed within 14 days after the balloon injury and their common carotid arteries were fixed by perfusion with 4% (w/v) polyformaldehyde.

### 11. Measurement of luminal narrowing of the injured arteries

The common carotid arteries were fixed by perfusion with 4% (w/v) polyformaldehyde for 15 min under constant pressure. The fixed arteries were then removed, embedded in paraffin and sectioned at 5-µm thickness. Cross-sections were stained with hematoxylin and eosin. The neointimal and medial areas of each cross-section were measured with a light microscope (Zeiss AxioSkop 20) connected to an image analyzer, and the intima/medium (I/M) area ratio was calculated.

### 12. Immunohistochemical and immunofluorescence analyses

For each time point, corresponding tissue sections were deparaffinized. After quenching endogenous peroxidase with 0.3% H_2_O_2_, nonspecific binding was blocked by incubation in 10% normal goat serum (NGS) for all primary antibodies. After washing, primary antibodies were detected with a horseradish-peroxidase-labeled secondary antibody. For immunofluorescence, primary antibodies were detected with secondary antibody which is labeled by AlexaFluor 568 (Invitrogen). Cellular nuclei were stained with Hochest-33342 (1∶5000) (Invitrogen). Analysis was performed using a fluorescent microscope (Nikon, DXM1200F) with a computerized, digital image analysis system and Adobe PhotoShop software.

## Supporting Information

Figure S1 **Representative light micrographs of common carotid arteries from sham-, Veh-, sense-OPN, sp600125-, DMSO- and antisense-OPN treated rats at day 14 after balloon injury.** Arterial sections were stained with HE stain. a, Sham-operation; b,operation injury 14 days; c, operation+anti-sense OPN treated; d, operation+sense OPN treated; e, operation+sp600125; f, operation+DMSO. Scale bars represent 100 µm.
(TIF)
