# Supplementary figures and images for: Expression and Function of Osteopontin in Vascular Adventitial Fibroblasts and Pathological Vascular Remodeling
Source: PLoS One. 2011 Sep 19;6(9):e23558. doi: 10.1371/journal.pone.0023558 (PMC3176202; doi:10.1371/journal.pone.0023558)

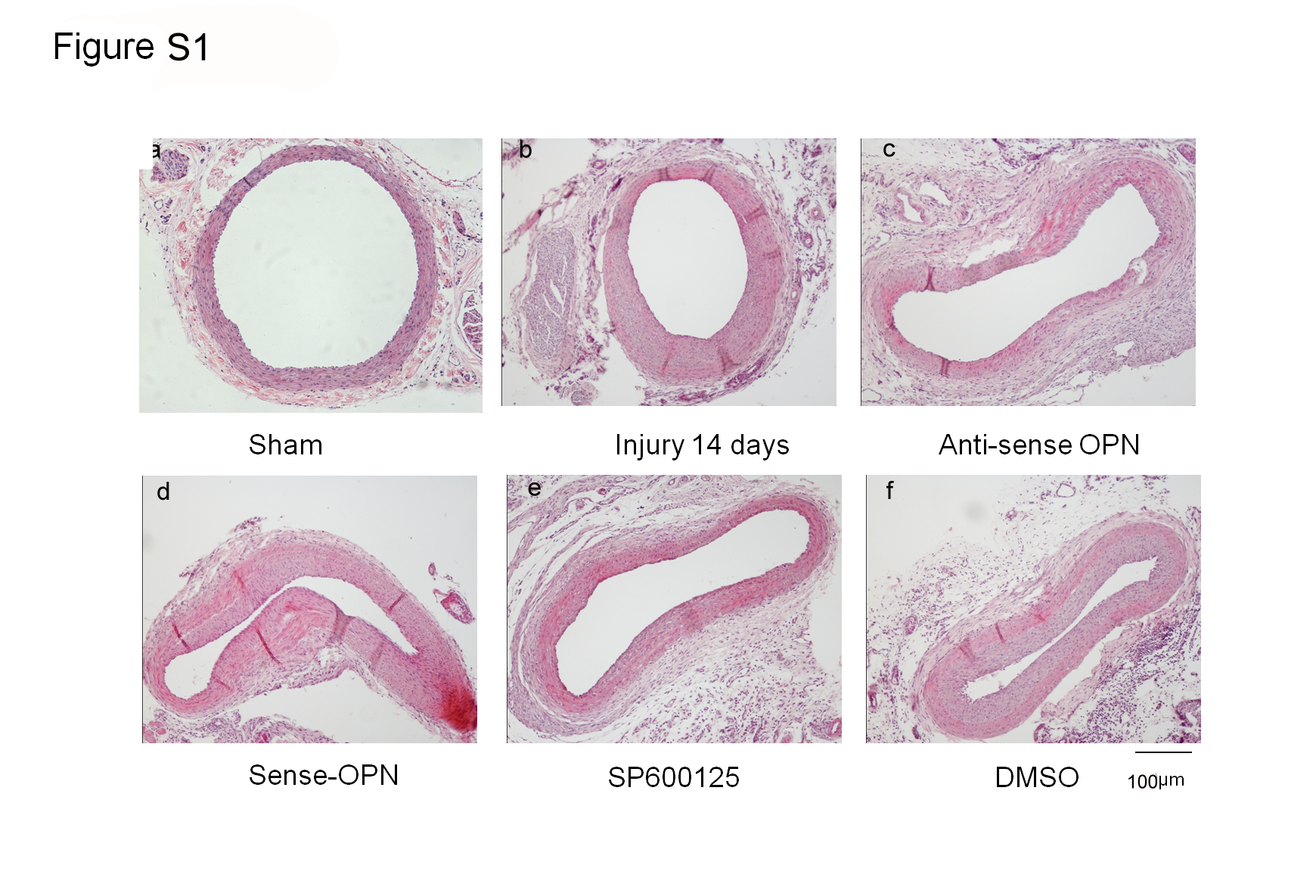

Supplement: Figure S1 — Representative light micrographs of common carotid arteries from sham-, Veh-, sense-OPN, sp600125-, DMSO- and antisense-OPN treated rats at day 14 after balloon injury. Arterial sections were stained with HE stain. a, Sham-operation; b,operation injury 14 days; c, operation+anti-sense OPN treated; d, operation+sense OPN treated; e, operation+sp600125; f, operation+DMSO. Scale bars represent 100 µm. (TIF) [file pone.0023558.s001.tif]
